# Models of inter professional working for older people living at home: a survey and review of the local strategies of english health and social care statutory organisations

**DOI:** 10.1186/1472-6963-11-337

**Published:** 2011-12-14

**Authors:** Claire Goodman, Vari Drennan, Fiona Scheibl, Dhrushita Shah, Jill Manthorpe, Heather Gage, Steve Iliffe

**Affiliations:** 1 Centre for Research in Primary and Community Care, University of Hertfordshire, Hatfield, AL10 9AB, UK; 2 Faculty of Health and Social Care Sciences, Kingston University and St George's University, Grosvenor Wing, Cranmer Terrace, London SW17 0RE, UK; 3 Social Care Workforce Research Unit, King's College London, Strand, London WC2 4LL, UK; 4 Department of Economics, University of Surrey, Guildford, GU2 7XH, UK; 5 Department. of Primary Care & Population Sciences, University College London, Royal Free Campus, Rowland Hill Street, London NW3 2PF, UK

## Abstract

**Background:**

Most services provided by health and social care organisations for older people living at home rely on interprofessional working (IPW). Although there is research investigating what supports and inhibits how professionals work together, less is known about how different service models deliver care to older people and how effectiveness is measured. The aim of this study was to describe how IPW for older people living at home is delivered, enacted and evaluated in England.

**Method:**

An online survey of health and social care managers across England directly involved in providing services to older people, and a review of local strategies for older people services produced by primary care organisations and local government adult services organisations in England.

**Results:**

The online survey achieved a 31% response rate and search strategies identified 50 local strategies that addressed IPW for older people living at home across health and social care organisations. IPW definitions varied, but there was an internal consistency of language informed by budgeting and organisation specific definitions of IPW.

Community Services for Older People, Intermediate Care and Re-enablement (rehabilitation) Teams were the services most frequently identified as involving IPW. Other IPW services identified were problem or disease specific and reflected issues highlighted in local strategies. There was limited agreement about what interventions or strategies supported the process of IPW. Older people and their carers were not reported to be involved in the evaluation of the services they received and it was unclear how organisations and managers judged the effectiveness of IPW, particularly for services that had an open-ended commitment to the care of older people.

**Conclusion:**

Health and social care organisations and their managers recognise the value and importance of IPW. There is a theoretical literature on what supports IPW and what it can achieve. The need for precision may not be so necessary for the terms used to describe IPW. However, there is a need for shared identification of both user/patient outcomes that arise from IPW and greater understanding of what kind of model of IPW achieves what kind of outcomes for older people living at home

## Background

The challenge faced by health and social care services in the developed world is to create integrated systems that address frailty [1-3]. Models of long-term chronic disease management for frail older people emphasize the need for multi-professional, pan-agency collaborative working that promotes closer working between health and social care organizations (e.g.[4-7]). At an organsational level this may be achieved through a range of methods, including, joint funding, networks of care, co-location or focusing on a single problem or issue. Less is known about how this is achieved by different models of service delivery that encompass inter professional working (IPW). This paper reports on a survey and document review of IPW for community dwelling older people in England that aimed to establish how IPW is delivered, enacted, and evaluated.

In England, government policy exhorts health and social care services and practitioners to work together in the support of older people at home (Department of Health (DH) [8-14]). Each of these policies assumes that integrated/partnership working can improve access to care, reduce costly duplication and fragmentation of services, and ultimately have positive outcomes and cost consequences for the person, their family, and publicly funded health and social care services [1] An extensive literature on the strategies and frameworks supports inter professional working (IPW) within acute hospital or whole systems of care (e.g. [15-19], (Trivedi, D.G. et al, The effectiveness of Inter-professional working for older people living in the community: A systematic review Unpublished report Centre for Research in Primary and Community Care University of Hertfordshire). Studies of IPW for older people living at home focus, either on how different professionals co-ordinate care across health and social care services or test models of service delivery designed to help different professionals identify people at risk to reduce unplanned hospital admissions or moves to long-term care (e.g. [20-23]). In England, a multiplicity of service models has evolved for this population within and between health and social care organizations. IPW is a useful umbrella term that has been used since 2000 to describe different ways of working that support integration within and across health and social care organisations [24]. Whilst there is an increasingly sophisticated theoretical understanding of what supports IPW [19], less is known about the ways these different models of IPW provide health and social care at the patient/user level or how effectiveness is evaluated.

## Methods

Two different approaches were used to capture the range of approaches to IPW adopted by statutory health and social care organisations. The first was a survey of health and social care managers directly involved in providing services to older people. The second was a review of local strategies for older people's services published by those with statutory responsibilities: primary care organisations (NHS Trusts) and local government adult services (social services).

Together these aimed to provide a national picture at an operational level of what was meant by IPW and how effectiveness is defined and measured.

### Development of the survey

An online survey tool was developed for managers. The questionnaire's content was informed by three sources of information: findings from a systematic review of IPW for community dwelling older people (Trivedi, D.G. et al, The effectiveness of Inter-professional working for older people living in the community: A systematic review Unpublished report Centre for Research in Primary and Community Care University of Hertfordshire) a selective review of relevant theoretical literature [25-30]; and findings from in-depth exploratory interviews with 10 purposively selected managers/team leaders with a focus on older people, working in NHS and local authority adult services and third sector or voluntary organisations. These combined sources provided an overview of the evidence of effectiveness for IPW, different models of IPW used with community dwelling older people, and clarification at a time of organisational change, of the language and organization framing of IPW across health and social care services.

The online survey had 17 questions. These covered the range of services for older people that involved IPW and how IPW was organized. Respondents were then asked to identify the two services involving IPW that they knew most about and answer more detailed questions about these. The questions addressed organisation and management of IPW, professionals involved, and sought information on patterns of referral and communication: resources used; outcome measures and user involvement in service evaluation. Finally, respondents were asked about the impact and contribution of IPW and how it was evaluated in their organisation. The questionnaire was piloted with 20 health and social care professionals or managers. Following their input, the survey was simplified and more questions were included that could offer the option of free text replies. The survey took 15 to 20 minutes to complete.

The target population for the survey was managers with operational responsibilities for the provision of services to community dwelling older people in the 152 Councils with Adult Social Services Responsibilities (CASSRs) and 150 NHS Primary Care Trusts (PCTs) in England. In England the aim of adult social services provided by CASSRs is to enable people to live independent lives in the community as far as possible, this is done through signposting to relevant organizations, individual assessment and/or publicly funded provision or commissioning of services for people who meet mainly low income eligibility criteria. At the time of the study Primary Care Trusts (PCTs) were responsible for both the local area NHS budget (commissioned both primary and secondary care) and also the provision of community health services (free at the point of delivery) in 'provider' arms of their organizations.

Identification and introductions to relevant managers were facilitated through the Association of Directors of Adult Social Services (ADASS) and the eight regional offices of the National Institute for Health Research Primary Care Research Network. The survey protocol was reviewed and approved by the University of Hertfordshire health and social care research ethics committee. The NHS research ethics service judged the survey to be a service evaluation.

All survey responses, including incomplete responses, were collated. Participants did not answer all fields, so the total number of responses for some questions varied. Descriptive statistics were used to summarize the survey results. Free text responses were analysed using content analysis[31].

### Local strategy Review

To supplement the survey data and provide an organsational perspective on how IPW was commissioned, provided and evaluated for this population a review of local strategies or plans for older people services was undertaken.

The method of documentary analysis [32] drew on the principles of systematic review methodology [33]. This included: document retrieval, review and scrutiny by two researchers, information retrieval using a data extraction sheet and analysis against the research objectives. Public domain, published and current Local Area Joint Older People Strategies were sought using internet search engines across nine government regions. Search terms included 'older people joint strategy', 'older people strategy', 'older adult strategy', and 'joint commissioning for older people' joint commissioning. Email requests were made to named individuals if a version was not available to download or not apparent on the web site. Following consultation with older people's representatives and the study advisory group, searches were extended to include Strategies for older people with Mental Health Problems and Strategies to support Carers. Data extraction focused on:

• The language of IPW between organisations, services and at the professional/service user level

• The identified types and mechanisms of IPW at organisation, service and service user level for older people who require support and care from health and social care organisations

• Performance targets and any service user outcomes

• Evidence of older people's input in evaluation and performance monitoring.

Information related to services, commissioning and plans for the promotion of healthy ageing, general well-being or social inclusion was excluded unless there was specific reference to IPW.

The findings presented below from the survey and documentary review are organized to reflect the common themes that arose from the two data sources.

## Results

**Figure 1:**
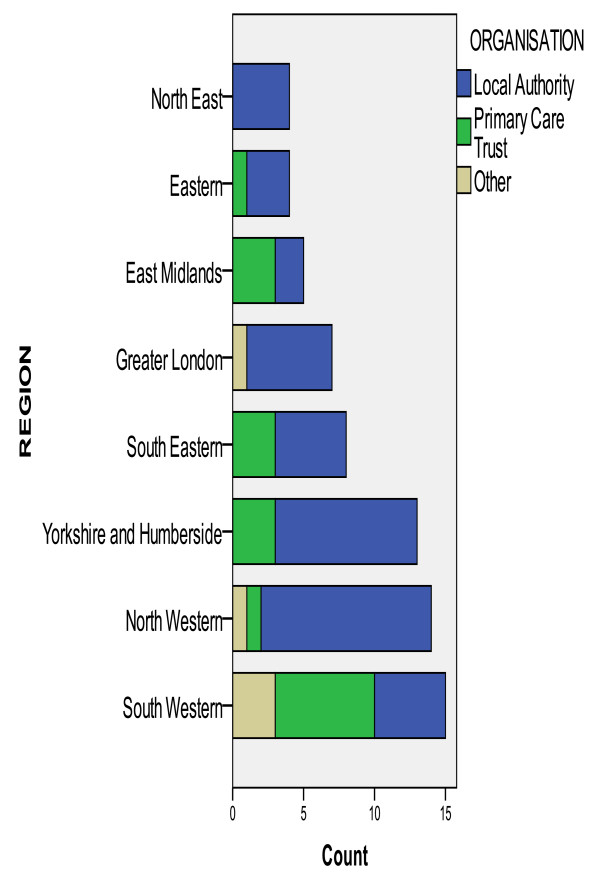
**Organisation by regional location**.

The online survey was circulated to health and social care professionals/managers in 292 organisations (142 Local Authorities (LAs), 150 Primary Care Trusts (PCTs)). There were 91 responses, a response rate of 31 percent comparable with other similar surveys [34]. Figure 1 summarises the pattern of response.

The search strategies identified 50 local documents specific to IPW for older people living at home across health and social care. Thirteen of the documents were published only in the name of the LA although each stated that consultation had occurred with relevant other organisations, such as PCTs.

### Language and Definitions of Inter Professional Working

The term "interprofessional working" although widely used in the academic literature was not recognized or used in the survey responses or documents reviewed. There was a hierarchy of definitions of terms surrounding IPW within organisations. Key phrases and terms were used to differentiate between IPW provision at different levels. These were not transferable across organisations but there seemed an internal logic to how key phrases and terms were used by different organisations and managers. In strategy documents, the term used to capture IPW at an organizational and service level most frequently was "partnership working". In contrast, the term used most frequently in the description of IPW at the professional and service user level was "joined up services". This was apparent even though other terms could also be used such as "joined up working," "joined up services", "joint working", "integrated working", "multi-agency working", "multi-disciplinary working" and "integrated health and social care".

**Figure 2:**
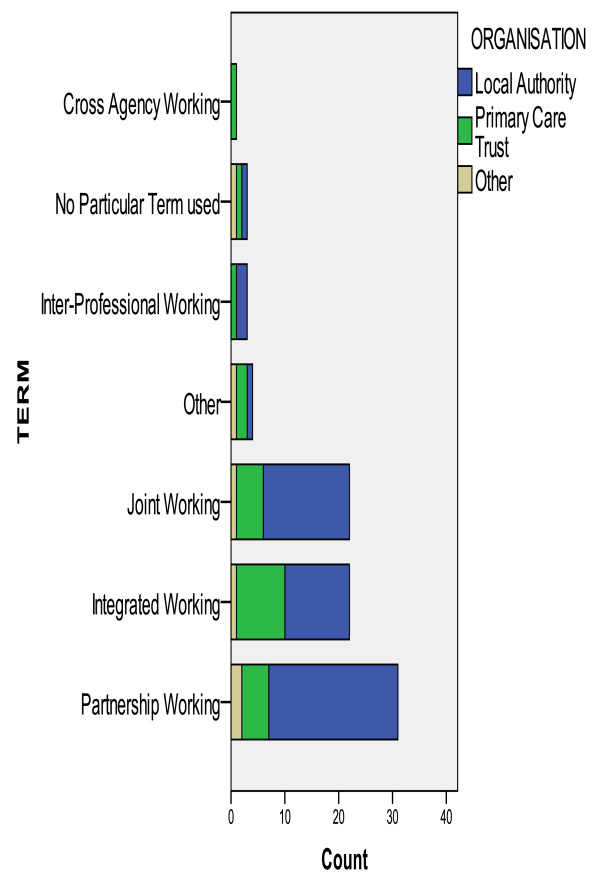
**Range of terms used to describe Interprofessional working by organisation**.

This finding was echoed in the survey responses about how IPW was defined within organisations. There was no consensus that different phrases or terms referred to specific levels of IPW organisation. NHS respondents tended to favour the term "integrated working", whilst social care respondents used the term "partnership working". Figure 2 summarises the range of terms used by organisations to capture IPW for older people.

However, it was the free text responses that highlighted the differences in emphasis. It appeared that structural and cultural processes within an organisation could give rise to different terms being used to differentiate how IPW was understood in (but not between) organisations:

Seems to be different terminology depending on where staff are in the organisation - senior managers talk about integrated/aligned care, staff at front line talk about partnership working' (PCT manager)

There was also some evidence that legislation (section 75 agreements under the Health and Social Care Flexibilities of the NHS Act 2006 (originally S31 of the Health Act 1999) was informing how different terms relating to IPW were being used. A manager of LA Adult Social Care Services identified internal consistency in how IPW was described within her organisation, but, in contrast to the above PCT manager quoted, made reference to "partnership" as meaning strategic working and "joint working "as meaning service delivery:

'There is more than one term used pending the circumstances. For strategic commissioning we tend to use ''partnership'' or collaborative". For operations the most used terms are '' joint'' or ''integrated''. Sometimes the legal status of the arrangement will determine the word used for example with section 75 agreements'^1^

LA manager* (Section 75 of the NHS Act 2006 is a power available to NHS and local authority bodies. It makes provision for them to: undertake each other's functions, i.e. in commissioning or provision create pooled funds. E.g for commissioning from a single budget or to integrate the resources of provision, i.e. some or all staff and their functions to be merged and delivered from within a single pool of service).

The survey responses and internal consistency of language in the documents reviewed suggested there was some precision in how IPW services were represented for older people. This was applicable at the level of organisation of IPW, even if the terms used were not transferable and was site specific. However, as one respondent observed, language could be very fluid. When new initiatives, such as the proposed introduction of social enterprises [35] emerged the language changed to try and capture how this form of IPW might be different to what had gone before:

'...We also use the term collaborative particularly around End of Life care where some multi-agencies may merge into a social enterprise' PCT manager

### Interprofessional working within and between organisations

At strategic level, the document review found that joint commissioning strategies and joint commissioning groups were the most frequently mentioned mechanism for "partnership" between organisations. Some areas reported funding joint posts as a mechanism for integration, commissioning posts that covered NHS and LA adult care for older people, and some joint service managers at operational levels. The latter was a particular feature of mental health services for older people. There were also references to the use of pooled budgets (under the Health Act 2006 flexibilities) for joint equipment services and multi-disciplinary community mental health teams. Other examples found included the joint commission of a home bathing service and a joint health and social care team for older people. Joint planning and provider groups were also frequently cited - often in relation to the task of creating joint integrated pathways or integrated services models. These were invariably problem or issue specific e.g. "Falls Pathways" and "stroke pathways". Only one document described multiple pathways for the health and social care of older people.

### Range of services identified reliant on IPW and organizations involved

Most of the Strategies analysed reported current or planned joint or integrated services for the same types of function. This included the creation of a single point for information on health and LA services (to improve uptake of services) or the creation of single points of access to publicly funded services (excluding General Practice). Some highlighted the introduction of shared assessment and core electronic records. Joint or integrated teams existed in most areas. It was not always clear if this meant a variety of health professionals or included LAs professionals, such as social workers and LA occupational therapists. The types of teams most frequently referred to were: intermediate care, rapid response, collaborative care teams, re-enablement/community rehabilitation teams and those designed to address a specific need such as: falls prevention teams, stroke rehabilitation, early diagnosis and intervention teams for mental health problems, and end of life care.

In the survey, managers in both health and local authority adult services organisations identified similar IPW services referred to in the documentary review.

Community Services for Older People (97%) was the service most frequently identified as involving NHS and LAs working together. This referred to situations when health and social care professionals were jointly involved in the assessment and provision of ongoing care and support to older people living at home. Often this would involve the organisation of home care support, provision of living aids and equipment, and therapist and community nursing involvement. This, however was not the model of IPW that managers chose to describe in detail and was not referred to in the Strategies reviewed. Other IPW services identified by more than half of the respondents were problem or disease specific and reflected areas (falls prevention, stroke and end of life care) highlighted in the documentary review. Only eight managers identified Tele-care (or involvement in assistive technologies) as a mechanism to support IPW.

In the survey respondents were asked to distinguish between services that were reliant on IPW and those that required intermittent involvement by various professionals. IPW was always identified as a component of intermediate care (75%), NHS Continuing Care (NHS funding for people with very high or specialist care needs living at home or in care homes) (58%) while disease specific (Chronic Obstructive Pulmonary Disease 21% and Cardiac Rehabilitation 19%) services were the least likely to rely on different professionals working together (see Table 1).

**Table 1:** Services that involve IPW across health and social care; survey responses

Services identified as most likely to **Always **Involve Working with Professionals from other Organisations	
• Intermediate Care	

• Stroke Rehabilitation	

• Continuing Care	

• Community Services for Older People	

• Rapid Response Service	

• Re-enablement Teams	

• Falls Prevention	

The findings from both the review and the survey suggest that intermediate care is a universally recognised model of IPW that represents an embedded service across almost all NHS and local authority organizations in England. Intermediate care involves a range of health and social care professionals working together for a prescribed (short) period of time to either facilitate the transition from hospital to home or avoid admission to hospital. The objectives of care are to help the patient achieve functional independence and recovery of health as far as possible.

As has been found in previous evaluations of this approach (see Table 2), certain mechanisms supported IPW (agreed entry criteria, shared assessments, shared protocols, social care funding) but equally there was considerable variation in patterns of service delivery, location of care and numbers and types of professionals involved.

**Table 2:** IPW working: the example of intermediate care

**How many professionals work together?**:
Just under half of respondents reported that in their experience delivery of Intermediate Care involved more than 20 professionals working together.
**Referral**:
Intermediate Care services were most likely to be delivered through agreed referral pathways (39% - 47%) or eligibility criterion (32%) or some combination of the two (18%-28%).

**Information Sharing**:
In terms of Information sharing, professionals reported that they use shared assessments (9)-15.8%), 18 (31.6) said they used all methods of information sharing: i.e. Shared Assessments, Shared notes, Shared Care Plans, Shared learning. Most 22 (39%) said that they used 'some of these methods'. The questionnaire construction did not allow respondents to state which methods were used sometimes. But logically we can assume that the majority of professionals were using Shared Assessments (9 who chose that method plus the 18 who said they used 'all these methods').

**Communication**:
The most typical method of communication used to support/facilitate inter-professional working in the delivery of Intermediate Care was reported to be face to face meetings (39%-48%). The least used was email (9%-14%) while telephone communication was used by just over a quarter of professionals (32% -34%). The findings suggest that while face to face meetings were the preferred method for communication this method was used in conjunction with telephone and email.

**Decision Making**:
Decision making by protocol emerged as the most significant pattern of decision making for Intermediate Care (67%)

**Funding**:
Funding was most often organised under separate budgets for Intermediate Care (67%). It is worth noting that separate budgets was also highlighted as most typical (71%) of the structure for the other four services (Continuing Care for Older People, Falls Prevention, COPD and Re-enablement Services) identified by respondents as services they had knowledge and experience of.

**Patterns of Contact**:
The survey findings indicate that the level of contact professionals have with their client when delivering Intermediate Care was variable both in terms of the time-point in the care delivery (say in week 2 compared to week 20) and with regard to the type of professionals (social worker, housing officer, district nurse). Professionals did not practice any particular pattern of contact with clients but contact developed on individualised basis even when the service involvement was time limited.

### Evaluating effectiveness

All documents or Strategies reviewed reported consultation with older people in their development. None mentioned any specific plans for evaluation of IPW services, involving older people in performance review or what indicators might suggest if IPW was effective.

In the survey 42 (79%) respondents reported that their organisations undertook evaluations of IPW. The method most often used for evaluation was questionnaire based surveys (n = 20; 49%). Very few respondents reported built-in feedback systems (4) or organised discussions with user representatives (6).When asked to select between a range of indicators (reliability, continuity, access, no duplication, no conflict between professionals) there was no consensus about the best indicators of IPW.

The survey asked respondents to rate a series of statements on a rating scale that drew on the work of [26] and allowed them to make critical assessments of IPW (see Table 3). As the sample sizes were small and not all respondents completed all of the questions, it is not possible to draw out any differences in rating perceptions between LA social care and NHS managers.

**Table 3:** Responses to statements about interprofessional working

Rating Scale of Statements about Inter- Professional Working	
*In my experience inter professional working works best for particular groups of older people'*	**Strongly Agree 4 (14%)**
	**Agree 16 (57%)**
	**Disagree 6 (21%)**
	**Strongly Disagree 2 (7%)**

*'I think that inter professional working is an expensive way of proving support to older people at home'*	**Strongly Agree 0**
	**Agree 0**
	**Disagree 16 (44%)**
	**Strongly Disagree 18 (50%)**

*'Inter professional working can make the service seem more fragmented '*	**Strongly Agree 1 (3%)**
	**Agree 1 (3%)**
	**Disagree 12 (40%)**
	**Strongly Disagree 14 (47%)**

*Some professionals working inter professionally find it almost impossible to adapt how they work to fit with others*	**Strongly Agree 0**
	**Agree 9 (45%)**
	**Disagree 1 (5%)**
	**Strongly Disagree 5 (25%)**

*For inter professional working to be successful you need to have someone who is responsible for making everyone work together*	**Strongly Agree 10 (36%)**
	**Agree 11 (39%)**
	**Disagree 5 (18%)**
	**Strongly Disagree 1 (4%)**

However, it is possible to gain a sense of the importance and contribution of IPW. Some authors have suggested that there is a growing disillusionment with the rhetoric of IPW and partnership working [36]. At a service delivery level very few managers agreed with the critical statements that IPW creates more fragmentation and is an expensive way of supporting older people. Most agreed with the statements that IPW is essential to the provision of care for older people at home and that is suited to particular groups. Opinion was divided on the issue of whether informal working practices were more effective than formal work structures and if professionals could adapt their working practices to fit in with other professionals.

## Discussion

There is an enduring imprecision and ambiguity in the language and an increasing skepticism about IPW effectiveness [19,37]. However, the survey and documentary review revealed support for the concepts of IPW across NHS and LA managers. Findings consistently showed a) that IPW language is context dependent; b) the short-term focus and funding resources of many interprofessional service delivery models; and c) that few accounts including the perspectives of older people and their carers in the evaluation of interprofessional and integrated services.

The term IPW, despite its widespread use in the academic literature [38,39,18], was not used in organisational documents at strategic level or by managers. IPW encompasses a wide range of approaches to working across disciplines and agencies. Others have offered hierarchies of meanings and critiques of IPW that could help organisations structure and evaluate IPW (e.g. [40,41]). A key finding of the paper was that organisations created, over time, their own hierarchies or taxonomies of IPW. These were known to their members but not necessarily to those outside the organisation. There was greatest clarity and definition when IPW was shaped by funding streams and the introduction of new policies. There was mutual understanding of words and phrases that were tied to legal and financial agreements, but local practice seemed to foster localized and project-specific understandings of key terms across the NHS and LAs. With the increase in the use of personal budgets (cash for care[42]), and near universal commissioning of third sector and commercial providers to replace directly provided LA services in England [42], it is likely that the language of IPW will become even more imprecise, diverse and context specific. The need for precision may not be in the terms used to describe IPW but shared identification of user and patient outcomes that arise from IPW and what kind of IPW model of working achieves what kind of outcomes. From an organsational perspective, more attention needs to be given to the attributes of IPW rather than particular terms (that will always be subject to change).

In both the survey and the document review details about IPW for older people were provided from a narrow range of time-limited, problem-specific services, with intermediate care services the most frequently identified model of IPW. It is noticeable in the IPW literature that research similarly often focuses on time limited interventions or discrete user groups (e.g. children with special needs or people with mental health problems). It would seem that we know least about the impact of IPW for those older people, who, once they are in receipt of services, will have ongoing and changing needs that may draw on more than one model of IPW.

Services that had a more open-ended commitment to the care of older people and more diffuse goals did not feature as services of interest in the documentary review or the survey. This narrow depiction of services involving IPW for older people was perhaps indicative of the practical challenge of aligning the goals and working patterns of professionals employed across organisations when goals of care are open-ended and diffuse [1,19]. It is also possible that limited resources available to evaluate the impact of ongoing support for older people through IPW favours initiatives that are time limited. In addition, a focus on outcomes like avoidance of hospital admission and recovery from acute episodes of ill health is an approach that does not support continuity of care. Nor does it encourage a culture of IPW that organises itself around frail older people as opposed to a single event, disease or problem.

The findings show that it continues to be the case that a commitment to providing outcomes-focused services for older people is seldom carried into long-term home care services [43]. Furthermore, even when there are desired outcomes, the Audit Commission [44] found that formal funding arrangements to support IPW made little or no impact on reducing the number of older people who experienced adverse events, or on the length of time they spend in hospital for some common conditions.

There was reference to user involvement in the development and planning of IPW based services in the documents reviewed. However, we found no evidence of service user defined outcomes or examples of service user involvement in evaluation of different IPW models of care. It was difficult to establish how services that did not have a single issue/disease focus were organized, if there were shared accountability structures or how the effectiveness of IPW was defined across organisations. This focus on the implementation of health and social care services over evaluations of effectiveness for the patient or service user is well documented [19,38,43,45-47]. Despite our best efforts it was very difficult to identify who was best placed to describe IPW for older people even when taking account of the need for this to be spread between managers. Respondents spoke of the value of clear leadership for IPW, but, as not all respondents completed the survey, this could suggest that respondents did not have a clear framework for thinking about IPW. There was no consensus about mechanisms that supported IPW, indicators of effectiveness or the benefit of formal methods of IPW over informal practices that had developed over time.

### Limitations

The survey findings are limited by the response rate and the documentary analysis may not have been able to access relevant material, possibly because it was not in the public domain. However, the response rate compares favorably with similar studies conducted around the same time and the problem of partial completion of online surveys has been documented by others reporting on IPW/partnership working in health and social care [34,19]. There was a level of agreement between the findings of the documentary review and the survey which suggests that the range and scope of services that involves IPW for older people living at home were captured.

## Conclusions

This paper has captured how IPW for older people was represented, delivered and evaluated at organsational and professional levels. Health and social care organisations and their managers recognised the value of IPW, and there was a broad consensus that more could be achieved through IPW than not. However, at the point of service delivery, respondents were unable to comment on the detail or measures of effectiveness of IPW. This illustrates the complex mix of allegiances and contexts of care that influences how IPW is achieved at the different levels of service delivery [1,48]. At the patient or service user level of IPW, questions of what effectiveness might look like and when it was articulated, were framed by organsational preoccupations about resource use, rather than patient or user expectations.

The theoretical literature has demonstrated that it is possible to distinguish at an organsational level between the performances of different services working in partnership and also between various aspects of the performance [19,49,50]. There is a need to understand how different models of service delivery for older people living at home co-exist within the health and social care economy. The development of outcome measures that measure the impact of different service models of IPW on their recipients would enable service providers to differentiate between their long term and short term benefits and the effectiveness of one model of working over another.

In England the forthcoming Health and Wellbeing boards have been established as a forum for local commissioners across the NHS, public health and social care, and look set to involve elected representatives, with public involvement. Their remit is to discuss how to work together to improve the health and wellbeing of the people in their area. It is proposed that they will strengthen the democratic legitimacy of commissioning decisions, as well as providing a forum for challenge, discussion, and the involvement of local people. For older people's services, the findings from this survey and documentary review demonstrate the need to focus on the impact of IPW over time on recognised user specific outcomes (e.g. access to care, continuity of information, improved function, levels of frailty and so on). Further work on this could help inform future discussions between health and social care about what "good" looks like from both the services' and the older person's points of view.

## Competing interests

The authors declare that they have no competing interests.

## Authors' contributions

CG and VMD, drafted the paper; JM SI HG DS DS provided detailed comments on drafts and were involved in all stages of the study. CG, VMD designed and led the study. FS was involved in data collection, analysis and commented on earlier drafts of the paper. All authors read and approved the final manuscript.

## Pre-publication history

The pre-publication history for this paper can be accessed here:

http://www.biomedcentral.com/1472-6963/11/337/prepub
